# Palliative Care Nursing: Principles and Evidence for Practice

**DOI:** 10.1038/sj.bjc.6602371

**Published:** 2005-02-22

**Authors:** M Bisset

**Affiliations:** 1 Camden PCT, London, UK

The first edition of this book provides a comprehensive text for nurses working within palliative care, although it would also be an interesting addition to the bookshelves of other professionals associated with this speciality. The target readership is palliative nurses working at an advanced practice level and the aim is to provide an intellectual challenge to engender critical thinking regarding clinical care of the dying rather than a ‘*how to do*’ manual. These may be rather grand aspirations, but are ably backed by an international list of contributors. In the main, they have hit their target as the book represents a genuinely fresh approach to writing in this area and has successfully synthesised for the first time disparate information. Overall, this makes for an interesting read and certain chapters, most notably in Part One – Encountering Illness, that document the contemporary history of nursing within this speciality will become increasingly referenced and much valued.

There are four parts to the book: Encountering Illness; Transitions into the Terminal phase; Loss and Bereavement; and Contemporary Issues, each containing an overview from the editors introducing the principles and theoretical issues followed by shorter chapters that concentrate on aspects of the section. The difference is that they have avoided the rather predicable layout of physical, psychological, spiritual and so on, in favour of an integrated approach to the organisation of writing that flows more readily with the actual transitions people experience at the end of life. This approach has much to recommend as it avoids the pitfalls of making the discussions around aspects of care seem dislocated.

Part One – Encountering Illness is the strongest section. It is both illuminating and original and explores what it means for people who become ill and face death to take up the social role of patient and the implications for those that become their carers. This is examined by presenting short chapters including an excellent historical analysis followed by an account of global palliation developments and discussion of how such nurses continue to shape their approach to care. An examination of developments where patients themselves have increasingly become engaged with planning care has much relevance to recent policy imperatives and helps to put this in context, as does a review of the evidence thus far for end of life care for those without cancer and the questions it raises around organisation of the delivery of care and appropriate access. Developing palliative care in hospitals, a unique piece of writing on illness transitions, and robust overview of the communication literature all applied in a relevant way to clinical care. Lastly, there was a weaker overview on assessment in the end of life care, which is more rudimentary and missed the current emphasis that nurses are providing in areas such as pain and extending roles to include physical examination.

Part Two concentrates on the Transitions into the Terminal Phase. Again the editors have the confidence to give adequate exposure to dying, unlike other palliative care textbooks that cover this in a brief single chapter. There is valuable writing here with clinical applicability. Two particularly good examinations of the spiritual dimensions of palliative care on the excluded at the end of life is undertaken. A fundamental weakness of the book is the depth of discourse around the ethical issues at the end of life. The brevity ensured that the chapter on ethical issues was not examined in sufficient depth, leaving the sterile and remote from the clinical reality that is rather more complex. An area of great relevance is the need for palliative nurses to be articulate and fully aware of issues surrounding euthanasia. This book will not help them obtain more than a cursory examination of the argument. Although euthanasia is illegal in the UK, the authors persisted to give a neutral emphasis to the arguments. The argument against it was intellectually weak, missing an adequate exploration around how palliative nurses can engage suffering positively, an issue only partly dealt with, in the section chapters. Nurses increasingly have a major part in determining capacity and discussing issues of futility at the end of life and this omission limits the comprehensiveness of the book. This text never claimed to emphasise aspects of particular symptom control apart from a useful chapter on emotions and cognitions and pain. I suspect this will therefore be more useful for palliative nurses teaching trainees rather than challenging clinical decisions surrounding pain, which advanced practice nurses currently make. Most palliative nurses therefore will still have to use this textbook as a companion to a palliative medicine textbook.

Part Three – Loss and Bereavement will be valuable to palliative nurses and many worry that their actions are self-taught and rarely make reference to theoretical underpinnings. Organ and tissue donation are deservedly dealt with and the chapter on families and children facing loss and bereavement is especially informative and will greatly enhance how nurses approach clinical care. A nurse's role in supporting a person in exploring funeral plans would have been a useful addition here. The last section on Contemporary Issues is well balanced and introduces discourse on professional boundaries and the cost of caring, then moving onto education, research, governance and leadership.

The editors are to be congratulated in presenting a concise corpus of contemporary relevant issues of nurses' roles in palliation. Those in the speciality working at advanced practice level should certainly own a copy. Most of the material presented does indeed raise questions, however, the authors had scope for some truly visionary thinking especially in areas of clinical practice, which they did not quite harness. For this reason, I do not think that in this first edition they have quite managed to reach the claim to test the intellect, which after all was the original premise. I look forward to the second edition.

